# Damage Associated Molecular Pattern Molecule-Induced microRNAs (DAMPmiRs) in Human Peripheral Blood Mononuclear Cells

**DOI:** 10.1371/journal.pone.0038899

**Published:** 2012-06-22

**Authors:** Sebnem Unlu, Siuwah Tang, E. na Wang, Ivan Martinez, Daolin Tang, Marco E. Bianchi, Herbert J. Zeh, Michael T. Lotze

**Affiliations:** 1 Department of Surgery, School of Medicine, University of Pittsburgh, Pittsburgh, Pennsylvania, United States of America; 2 Infectious Disease and Immunogenetics Laboratory, Department of Transfusion Medicine, National Institutes of Health, Bethesda, Maryland, United States of America; 3 Department of Molecular Biology and Functional Genomics, San Raffaele University and Scientific Institute, Milano, Italy; 4 Department of Genetics, School of Medicine, Yale University, New Haven, Connecticut, United States of America; University of Strasbourg, France

## Abstract

Endogenous damage associated molecular pattern molecules (DAMPs) released from necrotic, damaged or stressed cells are associated with an inflammatory response. Whether the microRNA (miR) expression signature of this response is different from that of a pathogen associated molecular pattern (PAMP)-stimulated inflammatory response is unknown. We report here that miR-34c and miR-214 are significantly expressed in fresh human peripheral blood mononuclear cells (PBMCs) exposed to DAMP-containing freeze-thaw lysates, or to conditioned media from serum-starved and glucose-deprived cells (p<6×10^−4^ and p<3.7×10^−3^), respectively. Interestingly, only miR-34c expression was differentially expressed in PBMCs exposed to freeze-thaw lysates or conditioned media from wildtype High Mobility Group B1 (HMGB1^+/+^) mouse embryonic fibroblast (MEF) cells, when compared to cultures exposed to lysates or conditioned media from HMGB1^−/−^ MEFs. miR-155 expression in these cultures was negligible, but was significantly expressed in PBMCs stimulated with Lipopolysaccahride (LPS) or most other Toll-like receptor (TLR) ligands, making it the prototypic “PAMPmiR”. Exposure to a damaged human colorectal carcinoma cell line lysate (HCT116) similarly resulted in increased miR-34c and miR-214 levels. When PBMCs were pre-transfected with anti-miR-34c and then exposed to lysate, expression levels of IKKγ mRNA, a putative target of miR-34c, increased, while protein levels of IKKγ in cultures transfected with a pre-miR-34c were abrogated. Levels of miR-34c expression (as well as pro-inflammatory cytokines, IL-1β and TNFα) decreased when PBMC cultures were briefly pre-incubated with the K^+^ channel (inflammasome) inhibitor, glybenclamide, suggesting that inflammasome activation is upstream of miR-34c expression in response to DAMPs. Our findings demonstrate that a specific microRNA expression signature is associated with the inflammatory response to damaged/injured cells and carries implications for many acute and chronic inflammatory disorders.

## Introduction

Intracellular factors released from stressed, necrotic, or damaged cells (or tissues) serve as endogenous danger signals or ligands, triggering several stress receptors, and leading to the activation of an innate immune response [1]. These pro-inflammatory factors are termed damage-associated molecular pattern molecules or DAMPs [2]–[4]. A prototypic DAMP, high mobility group box 1 protein (HMGB1), is a highly conserved chromatin-binding protein, which is passively released from necrotic cells [5]. Although HMGB1 is involved in nucleosomal stabilization and transcriptional regulation of gene expression [6], once released from stressed or necrotic cells, it leads to local promotion of autophagy, the recruitment of inflammatory cells and, with other factors, immune cell activation. Several other DAMPs released from injured or damaged cells are also pro-inflammatory and include the heat-shock proteins, S100 proteins, uric acid, genomic DNA, RNA, as well as ATP [7].

MicroRNAs (miRNAs) are endogenous, small, non-protein coding RNAs, of around 22 nucleotides in length [8]. Transcribed as long, primary RNA sequences, they are processed into precursor or pre-miR stem-loops of about 60 nucleotides in length through the nuclear-specific enzyme complex, which includes the RNAse III, Drosha, and its partner, DGCR8 [9]. The pre-miR is actively transported from the nucleus and further processed into a 21-nucleotide duplex. Via the RNA-induced silencing complex [10], the duplex is guided to bind target messenger RNAs (mRNAs) leading to repression of the target’s expression by inhibiting of translation or by targeting the mRNA for degradation or deadenylation [11]. In humans, up to one third of protein coding genes are predicted to be potential miRNA targets [11].

Here, we report that when human PBMCs are exposed to damaged HMGB1^+/+^ cell lysates, or conditioned media from serum-starved and glucose-deprived cells, both hsa-miR-34c and hsa-miR-214 are upregulated. However, in PBMCs exposed to HMGB1^−/−^ cell lysates, the levelsof hsa-miR-34c expressed are significantly less. We also demonstrate that one of the functional targets for miR-34c could be IKKγ an essential signaling intermediate of the NFκB inflammatory pathway. This data reveal a characteristic miR expression pattern of human inflammatory cells in response to cell damage or injury.

## Results

### miR-34c and miR-214 are Differentially Expressed in Human PBMCs Following Exposure to Damaged/necrotic Cell Lysates

To determine the microRNA expression signature in normal human PBMCs following exposure to sterile freeze-thawed lysates, we exposed four individual donor PBMCs either to MEF freeze-thaw lysates derived from HMGB1^+/+^ or from HMGB1^−/−^ cells (at 3×10^5^ cells/ml of PBMC culture). The optimal dose of DAMP-containing lysate was obtained earlier by assaying the TNFα released after exposure to increasing doses of lysates (Fig. S1A). Levels of other pro-inflammatory cytokines (IL-1β and IL-6) released from donor PBMCs also changed when exposed to the damaged/necrotic lysates (Fig. S1B). Additionally, TNFα released from HMGB1^+/+^ lysates was (to some extent) due to the presence of HMGB1 as pre-incubation of lysates with a neutralizing HMGB1 antibody at 10 ug/ml limited TNFα release from human donor PBMCs (Fig. S1C). Furthermore, pre-incubation with a blocking TLR2 antibody limited TNFα release when compared to cultures pre-treated with a control isotype antibody (Fig. S1D). TNFα release was diminished when cultures were exposed to boiled or hydrogen peroxide-treated HMGB1^+/+^ lysates (Fig. S2), indicating that the active DAMPs are proteins, which must have a native conformation and remain in a reduced state for activation of the inflammatory response.

**Figure 1:**
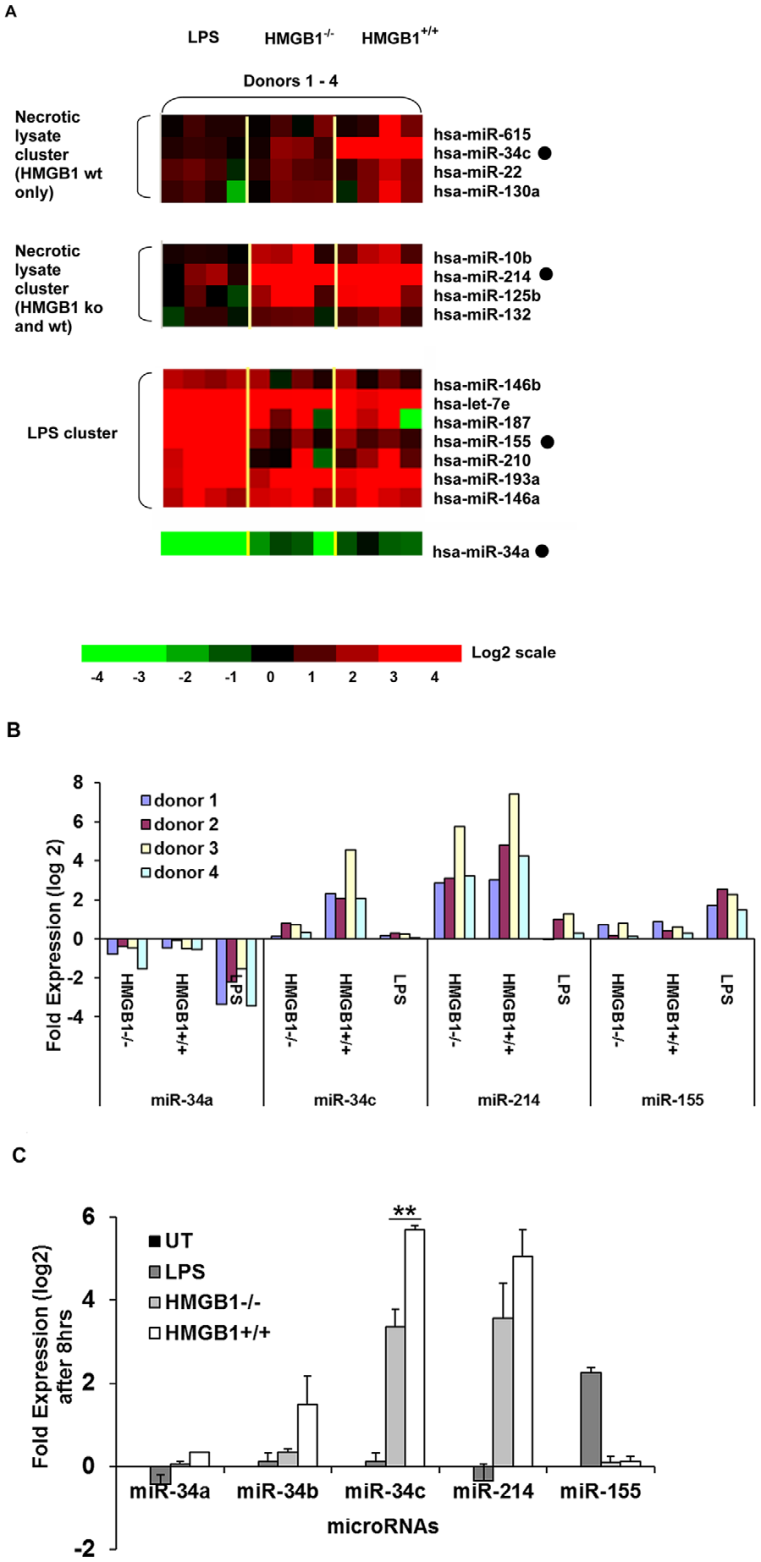
Expression of hsa-miR-34c and hsa-miR-214 is a hallmark of human PBMCs exposed to necrotic cell lysates. **A**: Hierarchical clustering or heat map of microRNA expression signatures (after real-time TaqMan RT-PCR array profiling) in donor PBMCs exposed to cell lysates and/or LPS. Total RNA extracted from PBMC cultures was run on microRNA TaqMan low-density arrays (TLDAs). **B**: Figure depicting changes in fold expression as log 2-transformed RQ (relative quantity) values of the statistically significant miRs (p values shown in Table 1) from each of the four donors, after exposure to the respective conditions. All values were calculated from 2^−δδCt^ (RQ values) where the endogenous control was snoRNA U48. **C**: Differential expression of hsa-miR-34a, miR-34b, miR-34c and other miRs when donor PBMCs are exposed to HMGB1^+/+^ or HMGB1^−/−^ lysates for 8 hrs. Data shown are average±s.d., of two independent experiments, each from a different donor, measured in triplicates, where ** indicates p<0.01, by paired Student’s t test.

For microRNA profiling, control cultures were either treated with LPS at 100 ng/ml, or left untreated for 48 hrs, respectively. Total RNA (with microRNA) was isolated and applied to low-density TaqMan PCR-based arrays (TLDAs), each array designed to detect 384 specific human microRNAs (GEO Accession Number, GSE37399). Relative quantification of miR expression was presented with respect to untreated cultures, and normalized to an internal RNA control, snoRNA U48. Statistically significant miRs were defined by an F-test among four treatment groups (n = 4/group) with p value <0.005. From this analysis, three differentially expressed microRNAs were revealed, namely, miR-214 (permutation p value 3.7×10^−3^), expressed specifically in human PBMCs exposed to either HMGB1^+/+^ or HMGB1^−/−^ lysates, and miR-34c (permutation p value 6×10^−4^), expressed in PBMCs exposed to HMGB1^+/+^ lysates alone (Table 1). Human PBMC cultures stimulated with 100 ng/ml of LPS revealed increased expression of miR-155 (permutation p value 4.2×10^−3^) as previously reported [12]. We also performed an F-test on the δCt values from all four treatment groups. This statistical analysis further revealed that miR-125b and miR-10b were upregulated in cultures exposed to either damaged lysate, while hsa-miR-34a expression was down-regulated in both LPS and lysate treated cultures (Table 2). Hierarchical clustering analysis of microRNA profiling confirmed that hsa-miR-34c is preferentially upregulated in PBMCs exposed to HMGB1-containing lysates but not HMGB1^−/−^ lysates (Figure 1A, upper panel). Hsa-miR-214 expression clustered with other miRs (such as miR-10b and miR-125b) when donor PBMCs were exposed to both types of cell lysate (Figure 1A, middle panel). Confirmed by our F-test analysis using δC_t_ values, hsa-miR-34a expression was down-regulated in all donor PBMCs after exposure to cell lysates or LPS, but relatively more so in LPS stimulated cultures (Figure 1A, lower panel). The LPS-induced microRNA signature was formed by a large cluster of miRs, including miR-155 and miR-187, as can be seen in the third panel, Figure 1A. Interestingly, hsa-let-7e, miR-146a, and miR-193a were over-expressed in both LPS and MEF lysate exposed PBMCs, indicating that they might represent the common miRs in DAMP- and PAMP-mediated inflammation. Together these data suggest that hsa-miR-214 expression is a general “DAMPmiR” expressed in human PBMCs exposed to damaged cells, while hsa-miR-34c is a miRNA that is sensitive to the presence of HMGB1 in damaged cells.

**Table 1:** Statistically significant miRs from microRNA profiling data after an F test of fold change in expression values.

microRNA	(KO^1^) Geometric meanof RQ^2^	(WT^1^) Geometric mean of RQ	(LPS) Geometric mean of RQ	p-value	
				Permutation	Parametric
**miR-155**	1.36	1.45	3.97	4.2×10^−3^	4.6×10^−4^
**miR-34c**	1.39	6.71	1.14	6×10^−4^	1.4×10^−3^
**miR-214**	13.27	28.9	1.56	3.7×10^−3^	4.8×10^−3^

1 necrotic cell lysates ^2^RQ is fold change in expression calculated from 2^−δδCt^.

**Table 2:** Statistically significant miRs from microRNA profiling data after F test of δC_t_ values.

Parametric p-value	FDR^1^	Permutation p-value	Geometric mean of δCt values in class 1^a^	Geometric mean of δCt values in class 2^b^	Geometric mean of δCt values in class 3^c^	Geometric mean of δCt values in class 4^d^	Unique ID
1.4e–05	0.005	<1e–07	13.0	9.9	8.7	13.6	miR-214
5.09e–05	0.01	0.0011	14.0	13.7	11.4	14.2	miR-34c
0.0005	0.07	0.0023	8.8	8.96	8.8	11.3	miR-193a
0.0007	0.07	0.0014	7.3	8.7	8.1	9.0	miR-210
0.001	0.08	0.0035	1.8	3.3	3.2	3.8	miR-155
0.0016	0.10	0.002	11.7	9.9	9.5	9.1	miR-34a
0.002	0.11	0.0031	7.1	8.0	7.6	9.6	miR-9
0.002	0.11	0.0026	1.7	2.2	2.2	2.6	miR-146b
0.003	0.14	0.0037	8.5	11.2	11.1	12.6	miR-187
0.004	0.15	0.0053	9.5	7.5	6.6	9.6	miR-125b
0.0048	0.17	0.0032	14.0	12.7	13.1	14.2	miR-10b-

1 False discovery rate.

a LPS-stimulated cultures.

b MEF HMGB^−/−^ lysate exposed cultures.

c MEF HMGB1^+/+^ lysate exposed cultures.

d Untreated cultures.

From the microRNA profiling data, fold expression values (as log 2–transformed RQ values) for the statistically significant microRNAs (hsa-miR-34a, miR-34c, miR-214, and miR-155) were calculated for each donor after exposure to lysates or LPS (Figure 1B). The fold expression changes for hsa-miR-34c in donor PBMCs exposed to HMGB1^−/−^ lysates varied from 0.1 to 0.78 fold, and 2.0 to 4.5 fold following exposure to HMGB1^+/+^ lysates. The fold expression changes for hsa-miR-214 varied from 2.8 to 5.7 in donor PBMCs exposed to HMGB1^−/−^ lysates, while in donor PBMCs exposed to HMGB1^+/+^ lysates, it varied from 2.9 to 7.3 fold. Fold expression changes of hsa-miR-155 in LPS-stimulated donor cultures varied between 1.45 to 2.55 fold. These findings were confirmed by measuring the levels of these miRs individually using TaqMan microRNA RT-PCR assays in two independent donors following exposure to damaged lysates or LPS. Figure 1C shows the fold expression changes (as log 2-transformed values) for hsa-miR-34a, miR-34b, miR-34c, miR-214 and miR-155 under these conditions. The fold increase in hsa-miR-34c expression was from an average of 3.4 fold (in donors exposed to HMGB1^−/−^ lysates), compared to an average of 5.7 fold (in donors exposed to HMGB1^+/+^ lysates).

### Differential Expression of miR-34c in Donor PBMCs Exposed to a Necrotic Human Carcinoma Cell Lysate

**Figure 2:**
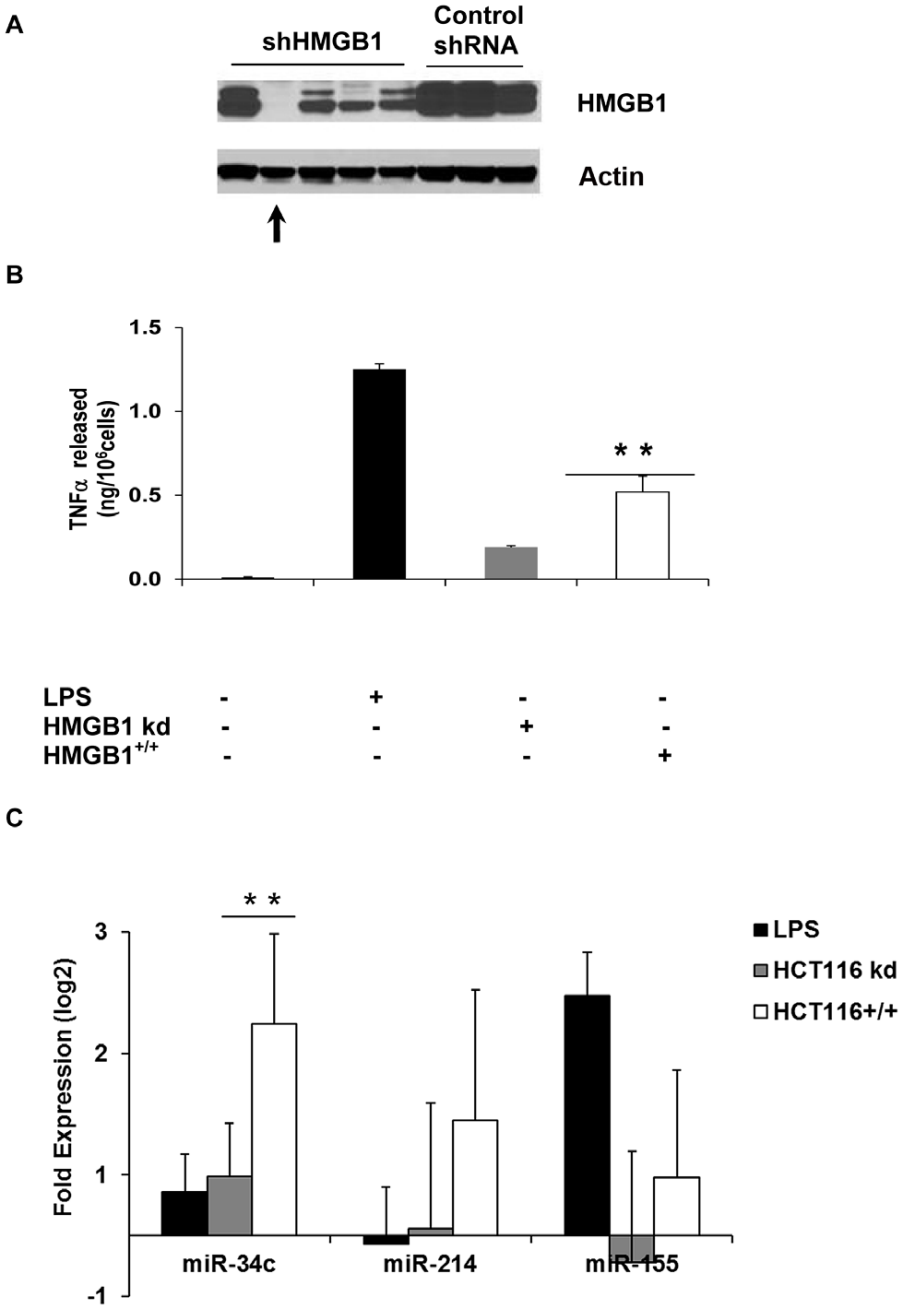
Differential expression of TNFα and hsa-miR-34c in human donor PBMCs following exposure to wild-type (wt) HCT116 or HMGB1 stable knock-down (kd) lysates. **A**: HCT116 cells were stably transfected with a shHMGB1 vector in the presence of Puromycin (100 ug/ml). The clone with complete knockdown of HMGB1 (indicated by the arrow) was chosen to make necrotic lysates. **B**: TNFα ELISA showing differential release of TNFα in human donor PBMC cell cultures following exposure to HCT116 lysates (wild-type, wt or HMGB1 knockdown, kd) for 24 hrs. The amount of HCT116 lysates used was 104 cells/ml of human PBMC culture. Data shown are the average±s.d. of three independent experiments, each from a different donor, measured in triplicates, where **indicates p<0.01, by Student’s t test. **C**: Fold changes in expression (as log-2-transformed RQ values) of hsa-miR-34c and hsa-miR-214 in donor PBMCs exposed to the indicated HCT116 necrotic lysates for 48 hrs. Values were measured using TaqMan real-time RT-PCR. Data shown are the average±s.d. of three independent experiments, each from an individual donor, measured in triplicates, where **indicates p<0.01, and where ***indicates p<0.001, by paired Student’s t test.

To exclude the possibility of confounding factors introduced by the use of xenogeneic fibroblasts, we next exposed normal donor PBMCs to necrotic freeze-thaw lysates from the human colorectal carcinoma cell line HCT116. Lysates were made from wild-type HCT116 and HCT116 that had been stably transfected with shRNA for HMGB1, respectively. Both TNFα release and hsa-miR-34c expression increased significantly following exposure to HMGB1^+/+^ lysates with respect to HMGB1^−/−^ lysates (Figure 2). As observed previously with necrotic lysates, hsa-miR-214 expression increased in donor PBMCs exposed to both types of HCT116 lysate. These results support the observation that both hsa-miR-34c and hsa-miR-214 are upregulated when human PBMCs are exposed to damaged or necrotic cells, where hsa-miR34c appears to be responsive to the presence of HMGB1.

### Expression of Hsa-miR-34c and Hsa-miR-214 does not Increase in Human PBMCs Stimulated with Specific Pathogen-activated Molecular Pattern Molecules (PAMPs) or TLR Ligands

**Figure 3:**
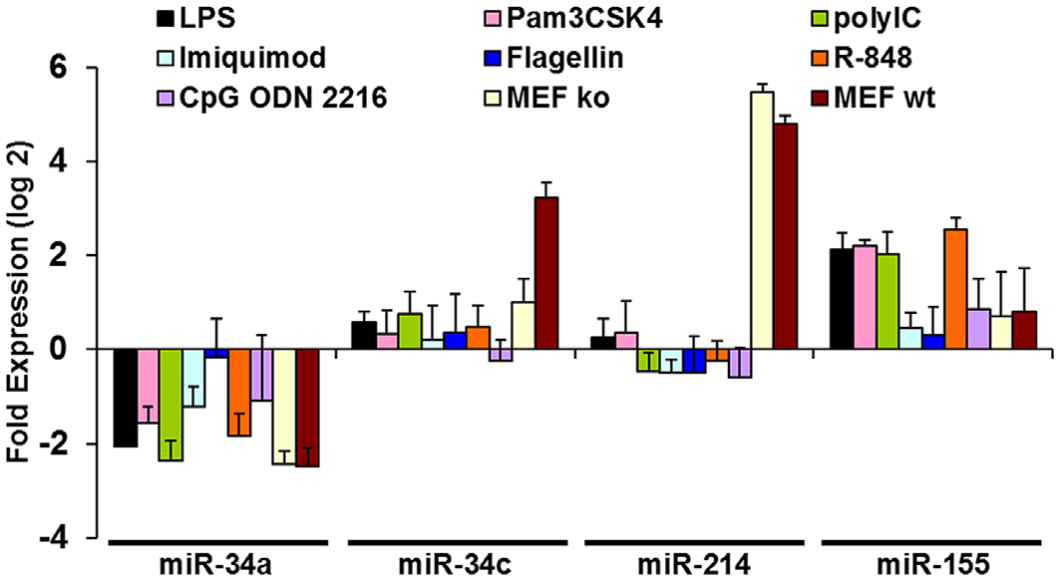
hsa-miR-34c and hsa-miR-214 are expressed at negligible levels in human PBMCs stimulated with various PAMPS or TLR ligands. Fold changes in expression of the indicated miRs in donor PBMCs (as log 2-transformed values) after stimulation with LPS (TLR4 ligand, 100 ng/ml), Imiquimod (TLR7 ligand, 1 ug/ml), CpG ODN 2216 (TLR 9 ligand, 1 ug/ml), Pam3CSK4 (TLR 2/1 ligand, 100 ng/ml), Flagellin (TLR 5 ligand, 100 ng/ml), poly I:C (TLR 3 ligand, 50 ug/ml), R-848 (TLR 7 and TLR 8 ligand, 1 ug/ml) respectively for 48 hrs and measured using TaqMan real-time microRNA RT-PCR. Data shown are average±s.d., of two independent experiments, each from a different donor, measured in triplicate.

To determine whether any PAMPs or TLR ligands are associated with miR-34c or miR-214 expression changes in donor PBMCs, we stimulated the cells with various PAMPS or known TLR ligands. Fig. 3 shows the fold expression changes (as log2-transformed values) of miR-34a, miR-34c, miR-214, and miR-155 after stimulation of donor PBMCs with various concentrations of TLR ligands. Expression of miR-34c and miR-214 was negligible in all samples stimulated with the various TLR ligands. However, the expression of miR-155 was common to many TLR ligand-stimulated PBMC cultures, especially those stimulated with LPS, poly I:C, Pam3CSK4, and R-848. These findings demonstrate that the “DAMPmiRs”, miR-34c and miR-214, are specific to PBMCs exposed to damaged cell lysates and that miR-155 may be considered a “PAMPmiR”.

### IKKγ is a Potential Functional Target of Hsa-miR-34c

**Figure 4:**
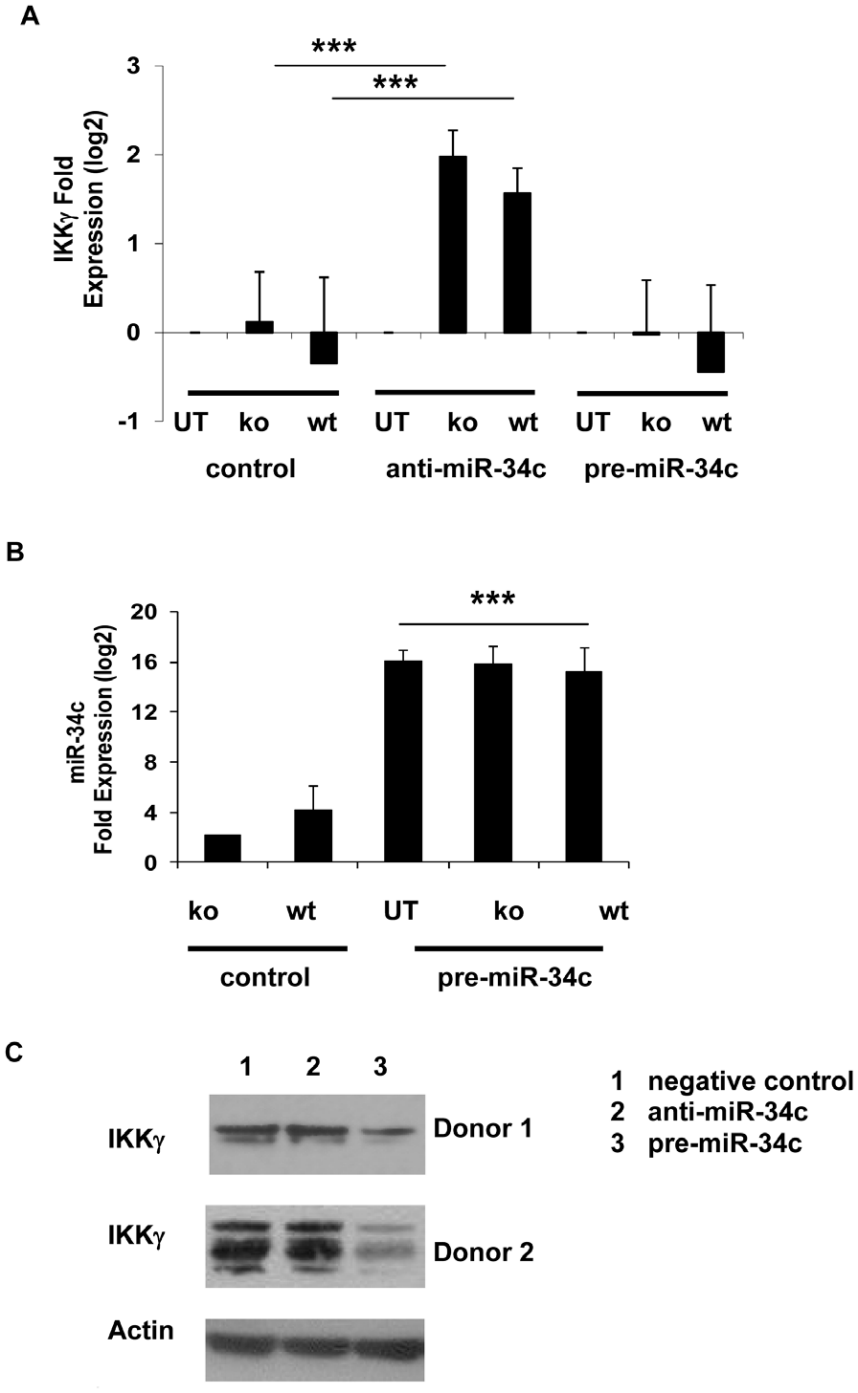
Changes in IKKγ mRNA and protein expression levels in human PBMCs pre-transfected with pre-miR-34c or anti-miR-34c and exposed to HMGB1^+/+^ or HMGB1 ^−**/**−^ **lysates.** **A**: Changes in fold expression (as log 2-transformed RQ values) of IKKγ mRNA levels in human PBMCs transfected with pre-miR-34c-5p or anti-miR-34c-5p and exposed to damaged HMGB1^+/+^ or HMGB1^−**/**−^ lysates for 8 hrs. Data shown are average±s.d., of two independent experiments and normalized to the untreated (UT) samples transfected either with control miR, pre-miR-34c or anti-miR-34c, where ***indicates p<0.001, by paired Student’s t test. **B**: Data shown are 48 hrs after transfection of pre- miR-34c and negative control precursor oligos into donor PBMCs. Values were measured using TaqMan real-time RT-PCR and normalized to sno RNA U48. Data shown are the average±s.d. of two independent experiments, each from a different donor, measured in triplicates, where ***indicates p<0.001, by paired Student’s t test. **C**: Decreased protein expression of IKKγ after transfection with pre-miR-34c (lane 3) or increased protein expression of IKKγ after transfection with anti-miR-34c (lane 2) and exposure to damaged HMGB1^+/+^ cell lysates for 24 hrs.

One of the computational targets in the Sanger Database (http://microrna.sanger.ac.uk/cgi-bin/targets/) for hsa-miR-34c is the regulatory non- enzymatic scaffold protein NEMO (NF-kappa B essential modulator also known as IKKγ (or Ik Kinase gamma). The computational binding energy level of hsa-miR-34c to IKKγ 3′ untranslated region (3′-UTR) is extremely low, about −22 kcal/mol (see Table S1), indicating the binding potential between the two sequences is very high. IKKγ is an important intermediate recruited in canonical (or classical) NF-κB signal transduction pathways [13]. It is activated upon stimulation of several DAMP or PAMP recognition receptors. We hypothesize that hsa-miR-34c may be required for fine-tuning expression of IKKγ, a key signal transduction intermediate in the expression of multiple immunity or inflammation associated genes. To investigate any changes in IKKγ levels in human PBMCs transfected with anti-miR-34c or pre-miR-34c and exposed to MEF lysates, IKKγ mRNA levels were assessed by real-time RT-PCR. Levels of hsa-miR-34 expression in pre-miR-34c transfected PBMCs were confirmed by the increase in fold expression of this miR using TaqMan microRNA real-time RT-PCR. There was an increase in fold expression from 2.1±1.97 to 15.9±1.85 (log2-transformed fold expression values) in cells pre-transfected with pre-miR-34c and exposed to HMGB1^−/−^ lysates (Fig. 4B), suggesting targeting of the seed sequence, preventing miR-34c-mediated degradation of IKK mRNA. As shown in Fig. 4A, transfection of anti-miR-34clead to a highly significant increase in IKKγ mRNA expression after exposure to either HMGB1^+/+^ or HMGB1^−/−^ lysates (RQ values calculated with respect to PBMCs transfected with negative control oligos and exposed to respective lysates). These findings support the notion that IKKγ may be a direct target of hsa-miR-34c. Shown below is the alignment of the miRNA sequence and the 3′UTR of IKKγ, with high level of complementarity at the seed region (in bold).
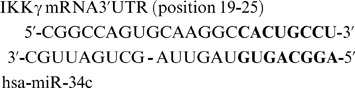



Interestingly, miR34c seed region sequence is highly conserved in humans and chimpanzees, as shown in Fig. S4, suggesting a possible major alteration in relatively recent evolutionary time.

We also evaluated the protein levels of IKKγ in PBMCs following pre-miR-34c or anti- miR-34c transfection and subsequent exposure to HMGB1^+/+^ or HMGB1^−/−^ lysates. Fig. 4C shows a significant reduction in the amount of IKKγ protein expressed in PBMCs pre-transfected with pre-miR-34c and exposed to HMGB1-containing lysates for 24 hrs.

### Levels of miR-34c and miR-214 Expression (and Pro-inflammatory Cytokine Release) Increased After Exposure of Donor PBMCs to Conditioned Media from Serum-starved and Glucose-deprived Cells

**Figure 5:**
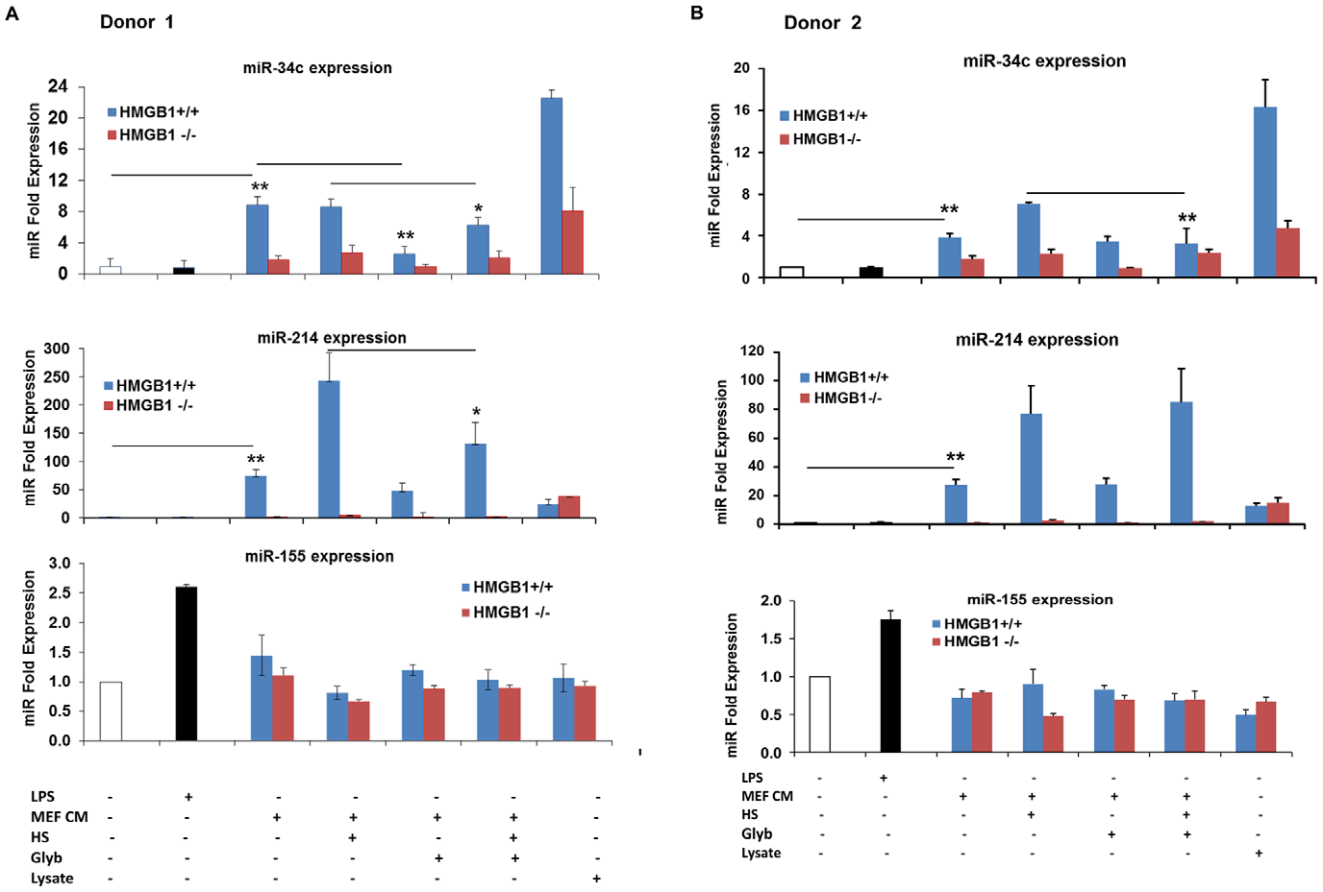
Expression levels of miR-34c and miR-214 are changed when donor PBMCs are exposed to conditioned media from dying cells. **A**: Changes in miR-34c, miR-214 and miR-155 expression in PMBCs (from one donor) exposed to conditioned media from HMGB1^+/+^ and HMGB1^−/−^ MEF cells. PBMCs were pre- incubated with 50 µM glybenclamide (Glyb) for 30 minutes before being exposed to conditioned media (MEF CM) for 48 hrs. Heat shock (HS) was carried out at 42°C for 2 hrs. Data shown are average±s.d. from one independent experiment in triplicate, normalized to untreated control samples and RNU48 as endogenous control. **B**: Changes in miR-34c, miR-214 and miR-155 expression in PBMCs from another donor exposed to conditioned media. Data shown are average±s.d. from one independent experiment in triplicate, normalized to untreated control samples and RNU48 as endogenous control, where **indicates p<0.01, *indicates p<0.05, by paired Student’s t test. The two respective groups compared in Figure 5 are for each miR expression graph, the treatment at the beginning of the straight line with the treatment at the end of the line. The control lane is the first treatment in each graph, which is indicated by the white bar.

Human donor PBMCs were exposed to conditioned media from serum-starved and glucose-deprived HMGB1^+/+^ or HMGB1^−/−^ MEF cells, as described in the methods. Total mRNA was isolated from donor PBMCs and Taqman miR PCR was carried out for miR-34c, mir-214, miR-155 and the endogenous nucleolar control RNA, RNU48. As shown in Fig. 5, both miR-34c and miR-214 were significantly expressed in cultures exposed to the conditioned media, compared to untreated cultures. However, miR-155 was expressed only in those cultures treated with LPS. Exposure of PBMCs to conditioned media with heat shock at 42°C further increased levels of miR-214 expression. Interestingly, levels of miR-214 were differentially expressed in PBMCs exposed to HMGB1^+/+^ or HMGB1^−/−^ conditioned media, but was not significantly affected when exposed to the respective lysates. It is plausible this difference may be due to the different modes of cell death under each condition leading to the release of different profile/levels of DAMPs in either of these conditions. Levels of the pro-inflammatory cytokines, IL-1β and TNFα were also increased in those cultures exposed to conditioned media (Fig. S3).

### Levels of miR-34c Expression (and Pro-inflammatory Cytokines) Decreased After Pre-incubation with the Inflammasome Inhibitor, Glybenclamide

When PBMC cultures were pre-incubated with 50 µM glybenclamide for 30 minutes, and exposed to conditioned media from serum-starved and glucose-deprived cells with heat shock, levels of miR-34c expression decreased significantly in both donor PBMC cultures (Fig. 5). This indicates that the inflammasome, shown to be activated when immune cells are exposed to necrotic/damaged cells [14], is important for the activation of miR-34c. Levels of pro-inflammatory cytokines (IL-1β and TNFα) also decreased after pre-incubation with glybenclamide (Fig. S3).

## Discussion

An inflammatory response to stressed or damaged cells/tissues occurs under various pathological conditions, including ischemia/reperfusion, bone fracture, aneurysm, autoimmune disease, and within the stressed/necrotic tumor microenvironment. Although there may be a clear lack of pathogenic infection in these conditions, the immune system is activated as a result of “misplaced self” molecules released from damaged/stressed cells. In this study we utilized HMGB1^−/−^ and HMGB1^+/+^ cell lysates to see whether the expression profile of inflammatory miRs in human PBMCs is different when exposed to either lysate.

It is interesting to note that the levels of expression of various DAMPs (such as heat shock proteins) are different in HMGB1^+/+^ cells compared to those without HMGB1 [15], indicating that HMGB1 plays a global transcriptional role in the expression of multiple genes, including genes for inflammatory proteins.

It is highly pertinent that microRNAs associated with the fine tuning of chronic sterile inflammatory pathways are elaborated. Knowledge of these miRs could be utilized as potential therapeutic and diagnostic tools in diseases associated with tissue injury and chronic inflammation.

In cancers that are not primarily associated with infection, chronic inflammation is an important factor in the promotion of tumorigenesis [16]. Interestingly, deletion of NEMO, an important regulator of NFκB signaling in inflammation, leads to heightened development of hepatocellular carcinoma [17]. Here, we demonstrate that NEMO is a functional target of an inflammation-associated miR, miR-34c.

Using a Taqman microRNA profiling low-density PCR array we identified several microRNA genes, including miR-34c, miR-214, miR-210, miR-125b and miR-10b in human PBMCs, which are involved in the inflammatory response to damaged cells. We show that miR-34c expression in human PBMCs is dependent on the presence of HMGB1 within cells serving as a source of lysates or conditioned media from stressed cells. We also demonstrate that the miRs induced in response to damaged cells are separate and distinct from those miRs upregulated in response to LPS, although some miRs (like miR-146a and let-7e) are common for both pathways. Increased expression of miR-34c has been reported in Duchenne Muscular Dystrophy, where muscle damage occurs at large scales [18], indicating that miR-34c may be a diagnostic biomarker of internal tissue damage.

One of the validated functional targets for miR214 is PTEN, a phosphatase and tensin homolog and a gene often deleted in many forms of cancer [19], [20]. High levels of miR-214 expression in human pancreatic tumors [20] have been observed, indicating that miR-214 may be a general marker of damage-associated inflammation, particularly in highly metastatic tumors etiologically associated with chronic inflammation, such as pancreatic cancer. It will be interesting to test whether the expression of miR-214 in inflammatory tumors is functional in promoting tumor growth.

High levels of miR-214 expression have been reported in a murine model of renal ischemia reperfusion injury [21]. This indicates that miR-214 may be a specific biomarker for internal tissue damage/injury. Some of the other “DAMPmiRs” that clustered together with miR-214 include miR-125b and miR-10b, where the latter can be involved in metastasis [22], [23]. Importantly, miR-125b deregulation has been observed in breast cancer [24].

It is interesting to note that many tumors have been associated with miR-155 over- expression [20]. This may be a specific marker for infection in that particular phenotype of cancer, and an indication that the source of chronic inflammation in these tumors is mainly due to infections. However, miR-155 expression has also been associated with the EMT (epithelial and mesenchymal transition) and invasiveness of cancer cells [25]. Down-regulation of miR-34a expression has been implicated in multiple cancers [26].

In our study, miR-34a was significantly down-regulated in human PBMCs exposed to damaged lysates, in contrast to miR-34c upregulation. Our findings indicate that miR-34c expression is due to the inflammatory response in human PBMCs and partly dependent on the presence of HMGB1 in cells from which damaged lysates or conditioned media were obtained. Interestingly, recent information suggests that deprivation of growth factors and deprivation of glucose differed in their signaling pathways [27] to promote autophagy. Possibly both HMGB1 and the miRs that we have identified may play a common role in driving autophagy and the response to inflammation [28]. Given the critical role of miRs in promoting the response to DAMPs, we also would speculate that cytokine stimulation of immune cells by potent agents such as Interleukin 2 [29], [30], [31] might also enhance autophagy as a global response to cell stress. Thus, microRNAs form another level of homeostatic control for the multiple gene networks involved in biological processes. It is therefore important to decipher which miRs are key switches that affect the variable phenotypes observed in health and disease. Our findings clearly indicate that miR-34c and miR-214 are specifically expressed in human PBMCs following exposure to sterile cell lysates or conditioned media from stressed cells, but not when exposed to PAMPs as TLR ligands. Hence, the design of agents targeting “DAMPmiRs” could be considered a potential therapeutic tool to limit aberrant sterile acute or chronic inflammation resulting from damaged or necrotic tissue.

## Materials and Methods

### General Statistical Analysis of Data

In brief, real-time TaqMan RT-PCR array profiling was performed in donor PBMCs exposed to cell lysates and/or LPS run on microRNA TaqMan low-density arrays (TLDAs). Various PAMPS or TLR ligands with fold changes in expression of individual miRs in donor PBMCs for 48 hrs were measured using TaqMan real-time microRNA RT-PCR. Changes in IKKγ mRNA and protein expression levels in human PBMCs pre- transfected with pre-miR-34c or anti-miR-34c and exposed to HMGB1^+/+^ or HMGB1^−/−^ lysates were evaluated. Expression levels of miR-34c and miR-214 were assessed in conditioned media from stressed (hypoxia, serum starvation) cells. Changes in miR-34c, miR-214 and miR-155 expression in PMBCs pre-incubated with 50 µM glybenclamide (Glyb) for 30 minutes before being exposed to conditioned media (MEF CM) for 48 hrs were used to assess the inflammasome pathway. Statistical analysis was carried out using 2 tailed, paired Student’s t test to compare two variables. The cut off p value for the Student t test was set at <0.05 level of significance.

### Isolation of Donor Human Peripheral Blood Mononuclear Cells (PBMCs)

Human PBMCs were isolated from normal donor buffy coats acquired from the Central Blood Bank, Pittsburgh, PA, in accordance with and approval by the University of Pittsburgh, Institutional Review Board. Ficoll-PaqueTM PLUS from GE Healthcare, Piscataway, NJ, USA (or Lymphocyte Separation Medium, from Mediatech Manassas, VA, USA) was used in a standard density centrifugation separation. Cells were re-suspended in IMDM, supplemented with 100 U/ml of Pencillin/Streptomycin and 10% (v/v) heat-inactivated fetal calf serum (FCS). For experiments, cells were seeded at 1×10^6^ cells/ml in 2 ml per well in 6 well plates.

### Cell lines & Reagents

Murine Embryonic Fibroblast (MEF) cells, which were either HMGB1^−/−^ or HMGB1^+/+^ were obtained from Dr. Marco E. Bianchi, Italy [32]. A human colon epithelial carcinoma cell line (HCT116) was obtained from Dr. Bert Vogelstein, Johns Hopkins University [33] and was stably transfected with HMGB1 shRNA (Sigma, St. Louis, MO) in the presence of 100 ug/ml puromycin (Invivogen, Carlsbad) to obtain a stable HMGB1 knockdown cell line. Polymyxin B was purchased from Sigma (St. Louis, MO). The HMGB1 antibody was a generous gift from Dr. Thomas A. Ferguson’s laboratory (St. Louis, MO). The TLR2 antibody was purchased from Abcam (Cambridge, MA). The TLR ligand set II (specific for human TLRs) was purchased from Alexis Biochemicals, EnzoLife Sciences International, Inc. (Plymouth Meeting, PA).

### Preparation of MEFs or Colorectal Cancer Cell Line HCT116 Freeze-thaw Lysates

Sub-confluent MEF or HCT116 cells were resuspended in ice-cold 1×PBS, with 1 mM PMSF and centrifuged at 10,000 g for 1 min at 4°C. The cells were re-suspended in cold non-denaturing lysis buffer (600 mM KCl, 20 mM Tris-Cl, pH 7.8 and 20% (v/v) Glycerol) at a concentration of about 100×10^6^ cells/1.3 ml of lysis buffer, supplemented with 1 mM PMSF, protease inhibitors and 1 mM DTT. The samples were dropped into liquid nitrogen until completely frozen and placed on ice to thaw slowly. When thawed, the samples were briefly vortexed at maximum speed. This was repeated 3 times. At this point, all cells were nonviable as determined by Trypan blue staining. The cell suspension was centrifuged at 10,000 g at 4°C for 10 minutes to pellet debris, and supernatants aliquoted into small volumes and frozen at −80°C. For an equivalent of 3×10^5^ cells/ml of cell lysates in culture, about 7 µl of the supernatant was added to 2 mls of PBMC culture in 6-well plates.

### Preparation of Conditioned Media and Treatment with Glybenclamide

2−3×10^6^ of MEF cells (either HMGB1^−/−^ or HMGB1^+/+^) were seeded in 20 cm diameter plates in 10% FBS in IMDM (supplemented with Pen/Strep) 2 days before adding RPMI without glucose. This resulted in about 9 million cells/cell type/plate after 2 days. 2 days later, the plates were washed twice with 1×PBS and 20 ml of RPMI without glucose was added to each plate. The cells were incubated for 24 hrs. After 24 hrs, the RPMI without glucose from each plate was collected, and centrifuged at 400 g for 5 mins. To 20 ml of RPMI without glucose, 10% FBS (v/v) (2 ml of stock serum), 0.2 ml of glucose solution (from 100 mg/ml stock) as well as 0.2 ml of Penicillin/Streptomycin stock solution was added. The conditioned media were stored on ice until it was added to 2 million of freshly isolated hPBMCs per well in a 6-well plate for 24 hrs for ELISA and 48 hrs for miRNA Taqman PCR. Heat shock was carried out at 42°C for an additional 2 hrs after incubation of MEF cells in serum and glucose-free RPMI. PBMCs were pre-treated for 30 minutes with 50 µM of Glybenclamide before incubation with conditioned media.

### Analysis of microRNA Profiling Data

The ABI Taqman SDS v2.3 software was used to obtain raw C_t_ values. The raw C_t_ values from each sample were converted to RQ or 2^−δδCt^ values. Briefly, δδC_t_ values were calculated from: (C_tsample_ – C_tendog._ _cont._) – (C_tuntreated_ – _Ctendog. cont._), log2 transformed and used for analysis. The endogenous internal control was the small nucleolar RNA U48, snoRNU48. Statistical analysis was performed using the F-test to determine the differentially expressed miRs with statistical significance and visualized by hierarchical clustering using Cluster™ and Treeview™ software. Statistical analysis was also performed on δC_t_ values, where the C_t_ value of the endogenous control, small nucleolar RNA U48, was subtracted from each sample’s C_t_ value.

### Real-time TaqMan RT-PCR for Specific microRNAs

Reverse transcription of specific microRNAs (from 10 ng of total RNA) was carried out using the RT-loop primers for each type of microRNA and the TaqMan microRNA RT kit from Applied Biosystems, according to instructions. The TaqMan primer part numbers from Applied Biosystems used for hsa-miR-34a, hsa-miR-34b, hsa-miR-34c, hsa-miR-155, hsa-miR-214, and RNU48 (snoRNA U48) were: 4373278, 4373037, 4373036, 4373124, and 4373085, respectively. cDNA obtained from this step was used to do real- time TaqMan PCR using the real-time primers provided on the ABI 7900HT Fast real- time PCR system (Applied biosystems), according to instructions. C_t_ values were converted to fold expression changes (RQ or 2^−δδCt^ values) following normalization to an internal small nucleolar RNA U48 (or snoRNA U48) and to the untreated (UT) control.

### Enzyme-linked Immunosorbent Assay (ELISA)

PBMC culture medium supernatants were collected 24 hrs after exposure to cell lysates, or conditioned media from serum-starved, glucose-deprived cells, or to 100 ng/ml Lipopolysaccharide (LPS, Serotype O55:B5, Sigma), or left untreated. Human TNFα ELISA kit (Pierce or BD Biosciences) or IL-1β ELISA (BD Biosciences) was used to assay the respective cytokines. For antibody treatments, lysates were pre-incubated on ice for 1 hour with antibody (10 µg/ml for HMGB1 antibody and 3.5 µg/ml for TLR2 antibody) before exposure to PBMC cultures. Culture medium supernatants were also assayed using the Luminex (Austin, TX) multiplex assay.

### Transfection of Human PBMCs with Pre-miR-34c or Anti-miR-34c Oligos

Donor PBMCs seeded at 15×106 cells/2 mls/well in 6-well plates were transfected with 5 nM (final concentration) of pre-miR negative control oligos (AM17110), miR-34c-5p precursor (PM11039), or anti-miR inhibitor oligos (AM11039), (Applied Biosystems, Foster City, CA), using siPORT Lipid transfection reagent (Applied Biosystems/Ambion, Austin, TX). These singles-stranded RNA-based inhibitors are chemically modified to increase stability and activity. After 48 hrs, the PBMCs were exposed to 3×10^5^ cells/ml of HMGB1^+/+^ or HMGB1^−/−^ cell lysate or left untreated for 24 hrs, and the PBMCs were lysed in lysis buffer (20 mM Tris base, 150 mM NaCl, 1 mM EDTA, 1 mM EGTA, 2 mM Na_3_VO_4_, 1% NP-40,10% Glycerol, pH 7.4) and probed for human IKKγ (molecular weight approx. 52 kDa) using antibody CST 2685 (Cell Signaling Technology, Beverly, MA).

### Quantitative Real-time RT-PCR for IKKγ mRNA

For quantification of IKKγ mRNA or hsa-miR-34c expression after 48 hrs of transfection, total RNA (with microRNA) was isolated using the miRNeasy mini kit (Qiagen) after exposure to damaged HMGB1^+/+^ or HMGB1^−/−^ lysates for 8 hrs. About 2.5 ug of total RNA was treated with DNase (TURBO DNA-free, Ambion) to remove any genomic DNA, and SuperScript III Platinum SYBR Green one-step real-time RT-PCR kit (Invitrogen) was used with primers specific for human IKKγ (SABiosciences, Frederick, MD). Fold expression (RQ values) levels were calculated with respect to β-Actin C_t_ values as an internal control.

### Isolation, Quantification and Quality Control of Total RNA with microRNA

The miRNeasy Mini Kit from Qiagen, (Valencia, CA) was used to isolate total RNA from each sample. Total RNA was quantified using a NanoDrop UV spectrometer and RNA integrity was determined using the 2100 Bioanalyzer (Agilent Technologies), with RNA 6000 Nano LabChip kit (Caliper Technologies) and RNA 6000 Reagents and supplies. Total RNA samples with an RNA integrity number of ≥8 were used for real-time PCR analysis.

### microRNA Profiling with TaqMan Low-density PCR Arrays (TLDAs)

384-sample TLDAs for microRNAs were used from Applied Biosystems. Explained briefly, 100 ng of total RNA was reverse transcribed according to ABI microRNA TLDA Reverse Transcription Reaction protocol. Samples were diluted 62.5 fold and loaded onto each port of the TLDA. The TLDA was loaded into the 7900 HT Sequence Detection system, and the default PCR program for TLDAs was used as directed in the instructions.

## Supporting Information

Figure S1 **Changes in pro-inflammatory cytokines released from human PBMC cultures exposed to damaged cell lysates.**
(DOCX)

Figure S2 **Changes in TNFα released from donor PBMC cultures exposed to boiled or hydrogen peroxide-treated HMGB1^+/+^ cell lysates.**
(DOCX)

Figure S3 **Changes in pro-inflammatory cytokines released from PBMCs exposed to conditioned media.**
(DOCX)

Figure S4 **Sequence alignment of miR-34c seed region in various species.**
(DOCX)

Table S1 **Some computational targets of hsa-miR-34c with very low binding energies.**
(DOCX)
