# Correction: Damage Associated Molecular Pattern Molecule-Induced microRNAs (DAMPmiRs) in Human Peripheral Blood Mononuclear Cells

**DOI:** 10.1371/annotation/18ec64ae-e086-446c-b63c-a71fa2bba046

**Published:** 2012-08-21

**Authors:** Sebnem Unlu, Siuwah Tang, E. na Wang, Ivan Martinez, Daolin Tang, Marco E. Bianchi, Herbert J. Zeh, Michael T. Lotze

The third author's name was spelled incorrectly. The correct name is: Ena Wang. The correct citation is: Unlu S, Tang S, Wang E, Martinez I, Tang D, et al. (2012) Damage Associated Molecular Pattern Molecule-Induced microRNAs (DAMPmiRs) in Human Peripheral Blood Mononuclear Cells. PLoS ONE 7(6): e38899. doi:10.1371/journal.pone.0038899

