# Supplementary material for: Damage Associated Molecular Pattern Molecule-Induced microRNAs (DAMPmiRs) in Human Peripheral Blood Mononuclear Cells
Source: PLoS One. 2012 Jun 22;7(6):e38899. doi: 10.1371/journal.pone.0038899 (PMC3382181; doi:10.1371/journal.pone.0038899)
Supplement: Figure S1 — Changes in pro-inflammatory cytokines released from human PBMC cultures exposed to damaged cell lysates. (DOCX) [file pone.0038899.s001.docx]

******

**+ HMGB1^+/+^ lysate**

**None HMGB1^-/-^ None Cont. Ab αHMGB1**

******

**C**

**B**

**A**

**D**

*******

**None HMGB1^-/-^ none Cont. TLR2 Cont. TLR2**

**Ab Ab Ab Ab**

**HMGB1^+/+^ lysate**

**Fig. S1 Changes in pro-inflammatory cytokines released from human PBMCs exposed to cell lysates.**
