# Supplementary material for: Damage Associated Molecular Pattern Molecule-Induced microRNAs (DAMPmiRs) in Human Peripheral Blood Mononuclear Cells
Source: PLoS One. 2012 Jun 22;7(6):e38899. doi: 10.1371/journal.pone.0038899 (PMC3382181; doi:10.1371/journal.pone.0038899)
Supplement: Figure S2 — Changes in TNFα released from donor PBMC cultures exposed to boiled or hydrogen peroxide-treated HMGB1+/+ cell lysates. (DOCX) [file pone.0038899.s002.docx]

*******

*******

*******

**HMGB1^-/-^ - + - - - - - -**

**HMGB1^+/+^ - - + boiled + + - -**

**H_2_O_2_ - - - - 36uM 3.6uM 36uM 3.6uM**

**Fig. S2 Changes in TNFα released from donor PBMC cultures exposed to boiled or hydrogen peroxide-treated HMGB1^+/+^ cell lysates.**
