# Supplementary material for: Damage Associated Molecular Pattern Molecule-Induced microRNAs (DAMPmiRs) in Human Peripheral Blood Mononuclear Cells
Source: PLoS One. 2012 Jun 22;7(6):e38899. doi: 10.1371/journal.pone.0038899 (PMC3382181; doi:10.1371/journal.pone.0038899)
Supplement: Figure S3 — Changes in pro-inflammatory cytokines released from PBMCs exposed to conditioned media. (DOCX) [file pone.0038899.s003.docx]

**A Donor X**

**LPS - + - -**

**MEF CM - - + +**

**MEF CM+HS - - - +**

*****

**B Donor Y**

**** ***

*****

**MEF CM + + + + + + - - -**

**HS - + - + - + - - -**

**Glyb - - + + - - - - -**

**DMSO - - - - + + - - -**

**Lysate - - - - - - + - -**

**LPS - - - - - - - - +**

**Fig. S3 Changes in pro-inflammatory cytokines released from PBMCs exposed to conditioned media.**
