# Supplementary figures and images for: Damage Associated Molecular Pattern Molecule-Induced microRNAs (DAMPmiRs) in Human Peripheral Blood Mononuclear Cells
Source: PLoS One. 2012 Jun 22;7(6):e38899. doi: 10.1371/journal.pone.0038899 (PMC3382181; doi:10.1371/journal.pone.0038899)

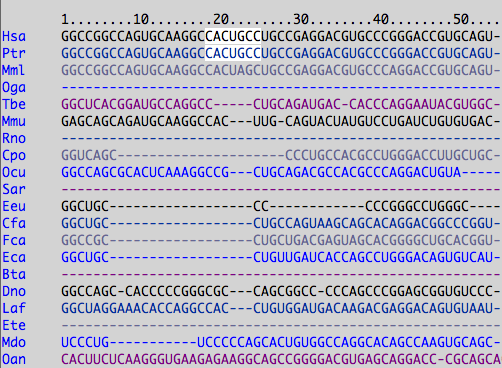


**Fig. S4 Sequence alignment of miR-34c seed region in various species.**

Supplement: Figure S4 — Sequence alignment of miR-34c seed region in various species. (DOCX) [file pone.0038899.s004.docx]
