# Supplementary material for: Damage Associated Molecular Pattern Molecule-Induced microRNAs (DAMPmiRs) in Human Peripheral Blood Mononuclear Cells
Source: PLoS One. 2012 Jun 22;7(6):e38899. doi: 10.1371/journal.pone.0038899 (PMC3382181; doi:10.1371/journal.pone.0038899)
Supplement: Table S1 — Some computational targets of hsa-miR-34c with very low binding energies. (DOCX) [file pone.0038899.s005.docx]

**Table S1. Some computational targets of hsa-miR-34c with very low binding energies**

| **Computational targets of hsa-miR-34c**  **(from Sanger database)** | | |
| --- | --- | --- |
| **Target mRNA** | **Binding Energy** | **No. of putative**  **miR-34c binding sites** |
| IKKγ (NEMO) | -21.98 | 25 |
| IKKε | -19.57 | 18 |
| MAPK13 | -18.99 | 16 |
